# Glial cell-line derived neurotrophic factor protects human islets from nutrient deprivation and endoplasmic reticulum stress induced apoptosis

**DOI:** 10.1038/s41598-017-01805-1

**Published:** 2017-05-08

**Authors:** Shadab Abadpour, Sven O. Göpel, Simen W. Schive, Olle Korsgren, Aksel Foss, Hanne Scholz

**Affiliations:** 1 Section for Transplant Surgery, Oslo University Hospital, Oslo, Norway; 2 Institute for Surgical Research, Oslo University Hospital, Oslo, Norway; 3 AstraZeneca R&D Gothenburg, Dept. CVMD Bioscience, Gothenburg, Sweden; 4 Institute of Clinical Medicine, University of Oslo, Oslo, Norway; 5 Department of Immunology, Genetics and Pathology, Science for Life Laboratory, Uppsala University, Uppsala, Sweden

## Abstract

One of the key limitations to successful human islet transplantation is loss of islets due to stress responses pre- and post-transplantation. Nutrient deprivation and ER stress have been identified as important mechanisms leading to apoptosis. Glial Cell-line Derived Neurotrophic Factor (GDNF) has recently been found to promote islet survival after isolation. However, whether GDNF could rescue human islets from nutrient deprivation and ER stress-mediated apoptosis is unknown. Herein, by mimicking those conditions *in vitro*, we have shown that GDNF significantly improved glucose stimulated insulin secretion, reduced apoptosis and proinsulin:insulin ratio in nutrient deprived human islets. Furthermore, GDNF alleviated thapsigargin-induced ER stress evidenced by reduced expressions of IRE1α and BiP and consequently apoptosis. Importantly, this was associated with an increase in phosphorylation of PI3K/AKT and GSK3B signaling pathway. Transplantation of ER stressed human islets pre-treated with GDNF under kidney capsule of diabetic mice resulted in reduced expressions of IRE1α and BiP in human islet grafts with improved grafts function shown by higher levels of human C-peptide post-transplantation. We suggest that GDNF has protective and anti-apoptotic effects on nutrient deprived and ER stress activated human islets and could play a significant role in rescuing human islets from stress responses.

## Introduction

Type-1 diabetes (T1D) results from autoimmune destruction of insulin producing pancreatic beta cells in islets of Langerhans, which is largely due to reactive T-cells^1^. The beta cells main function is to produce and secrete insulin to regulate the levels of glucose in the blood. Loss of beta cells function and mass increases the workload on the remaining fully functional beta cells^2, 3^. Consequently, these cells are more prone to experience endoplasmic reticulum (ER) stress and activation of unfolded protein response (UPR)^4^. Although short-term and mild activation of UPR secures proper folding of newly synthetized proteins in beta cells^5, 6^, prolonged and unresolved UPR activation triggers programmed cell death, which is associated with an increase in inflammatory cytokines and apoptosis through activation of caspase cascades^7, 8^.

Beta cell replacement by islet transplantation to selected patients suffering from T1D is currently becoming an established therapy^9^. However, its success rate is constrained by limited long-term islets graft survival partly due to massive loss of islets caused by hypoxia and nutrient deprivation in poorly vascularized islet grafts and inability of the islets to tolerate long-term stress environment^10–14^. The islet isolation procedure itself prior to transplantation also destroys cellular and non-cellular compartments of the pancreas, which potentially plays a role in islets loss and apoptosis^10, 12^. Newly isolated islets express high levels of ER stress sensors and activators (BiP, eIF2α, ATF4, sXPB1) as well as ER stress-associated apoptotic signals (JNK, CHOP, caspase3/7)^15^. Inositol-requiring enzyme1α (IRE1α) is also one of the UPR mediators triggering inflammation and induces transition from physiological to pathological UPR^5, 16, 17^. Culturing murine islets with growth factors such as insulin growth factor (IGF) or nerve growth factor (NGF) reduces ER stress and consequently ER stress induced apoptosis through activation of the PI3K/AKT signaling pathway^18, 19^.

Glial cell-line derived neurotrophic factor (GDNF) produced by glial cells plays an important role in the development of the enteric nerve system^20, 21^. GDNF signals through binding to GDNF-family receptor α-1 (GFRα-1) followed by GDNF-GFRα-1 complex binding to receptor tyrosine kinase (RET)^22–24^. Pancreatic beta cells share several biological characteristics with neuronal cells such as expression of neuronal transcription factors^25, 26^ and several findings link GDNF to beta cells survival and maintenance of beta cells function. Increased expression of GDNF has been reported in the proximity of pancreatic beta cells following islets injury suggesting involvement of GDNF in islets survival and repair^27^. Overexpression of GDNF in glial cells increases beta cell survival and improves glucose tolerance in transgenic mice^20^. *In vitro* pretreatment of human islets for 14 days in culture medium supplemented with human serum albumin, insulin growth factor -1 (IGF-1) and GDNF has also been shown to improve glycemic control and islet survival post transplantation in mice^28^. However, it is unclear whether or not GDNF can protect human islets against nutrient deprivation and ER stress induced apoptosis, which is detrimental in early phase after islet transplantation. By combining *in vitro* and *in vivo* approaches, we investigated the potential protective effect of GDNF on low-nutrient culture condition as well as ER stress induced apoptosis in human islets. Finally, we investigated the molecular signaling pathway by which GDNF protects against ER stress in human islets.

## Results

### GDNF improves function and viability of nutrient deprived human islets

**Figure 1:**
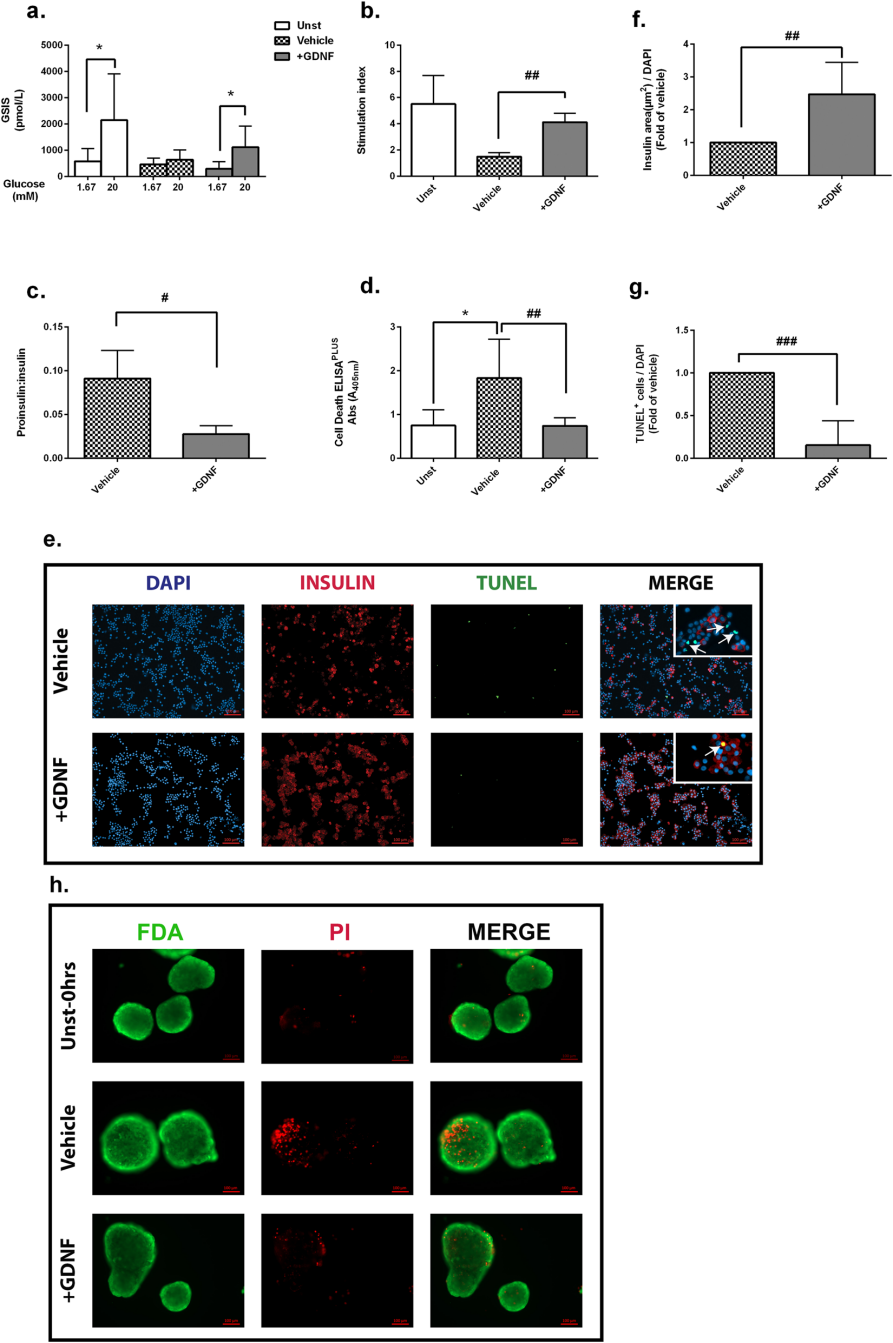
GDNF improves human islets function and viability under nutrient deprived culture condition. (**a**) Insulin secretion in response to basal (1.67 mM) and stimulated (20 mM) level of glucose (GSIS) (**b**) calculated as stimulation index in human islets prior to experiment start (0 hrs, unst islets) and after treatment for 72 hrs with or without GDNF (200 ng/nl) under nutrient deprived culture condition, n = 6. (**c**) Secreted insulin and proinsulin in culture medium were measured by EIA and presented as the proinsulin to insulin ratio, n = 4. (**d**) Apoptosis evaluated by cell death ELISA^PLUS^ in human islets, n = 6. (**e**) Representative images showing insulin (red) and TUNEL (green) with DAPI nuclear staining (blue) of dispersed human islets treated with or without GDNF. (**f**) Ratio of insulin area as well as (**g**) score of TUNEL^+^ cells over nuclear staining. Data is presented as a fold of vehicle islets, n = 3, Five images were taken from every slide and minimum of 2000 cells were scored. (**h**) Representative images showing intact islets stained for PI (red) and FDA (green) prior to islets culture (0 hrs) and after 72 hrs culture of nutrient deprived islets treated with or without GDNF. Statistical analysis: p-values were analyzed by Wilcoxon matched-pairs test in (**a**), nonparametric ANOVA with Dunn’s corrections in (**b**) (**d**), Mann-Whitney U-test in (**c**,**f**,**g**). For all analysis, data is presented as mean ± SD. *p < 0.05 vs.unst islets, ^#^0.05, ^##^p < 0.01, ^###^p < 0.001 vs. vehicle islets, *p < 0.05 vs stimulated (20 mM) glucose. Unst: unstarved islets. The n refers to the number of independent donors used for each experiment.

In order to investigate the effect of GDNF on islet function and survival under nutrient deprivation, isolated human islets were cultured for 72 hrs under low concentration of serum (0.5%) with or without GDNF. Unstarved islets cultured in media supplemented with 10% human serum was also included as control. To evaluate the islet function, we performed GSIS assay, and stimulation index (SI) was calculated as described in methods section. Insulin secretion in response to stimulated level of glucose was significantly increased for unstarved islets and nutrient deprived islets treated with GDNF, but not for vehicle (Fig. 1a). Therefore, nutrient deprived islets treated with GDNF performed significantly better compared to the vehicle (mean SI 4.10 ± 1.20 vs. 2.20 ± 1.50, p < 0.01) (Fig. 1b). To further evaluate the protective effect of GDNF on islets dysfunction, we measured the secreted levels of proinsulin and insulin in GDNF-treated islets compared to vehicle; (proinsulin: 1981 ± 247.90 vs. 2562 ± 413.40 pmol/L, insulin: 4324 ± 64.12 vs. 2381 ± 145 pmol/L) that revealed significant reduction in proinsulin to insulin ratio in GDNF-treated islets compared to vehicle (p < 0.05) (Fig. 1c). The improved functionality by GDNF on nutrient deprived islets was followed by reduced apoptosis compared to vehicle measured by DNA fragmentation using Cell Death ELISA^PLUS^ assay (0.74 ± 0.07 vs. 1.83 ± 0.36, p < 0.01) (Fig. 1d), TUNEL assay (Fig. 1e,g) and FDA/PI staining (Fig. 1h). In addition, immunofluorescent analysis showed a 2.0-fold increase of insulin staining in the GDNF-treated islets compared to vehicle (Fig. 1e,f).

### GDNF protects human islets from ER stress and consequently ER stress induced apoptosis

**Figure 2:**
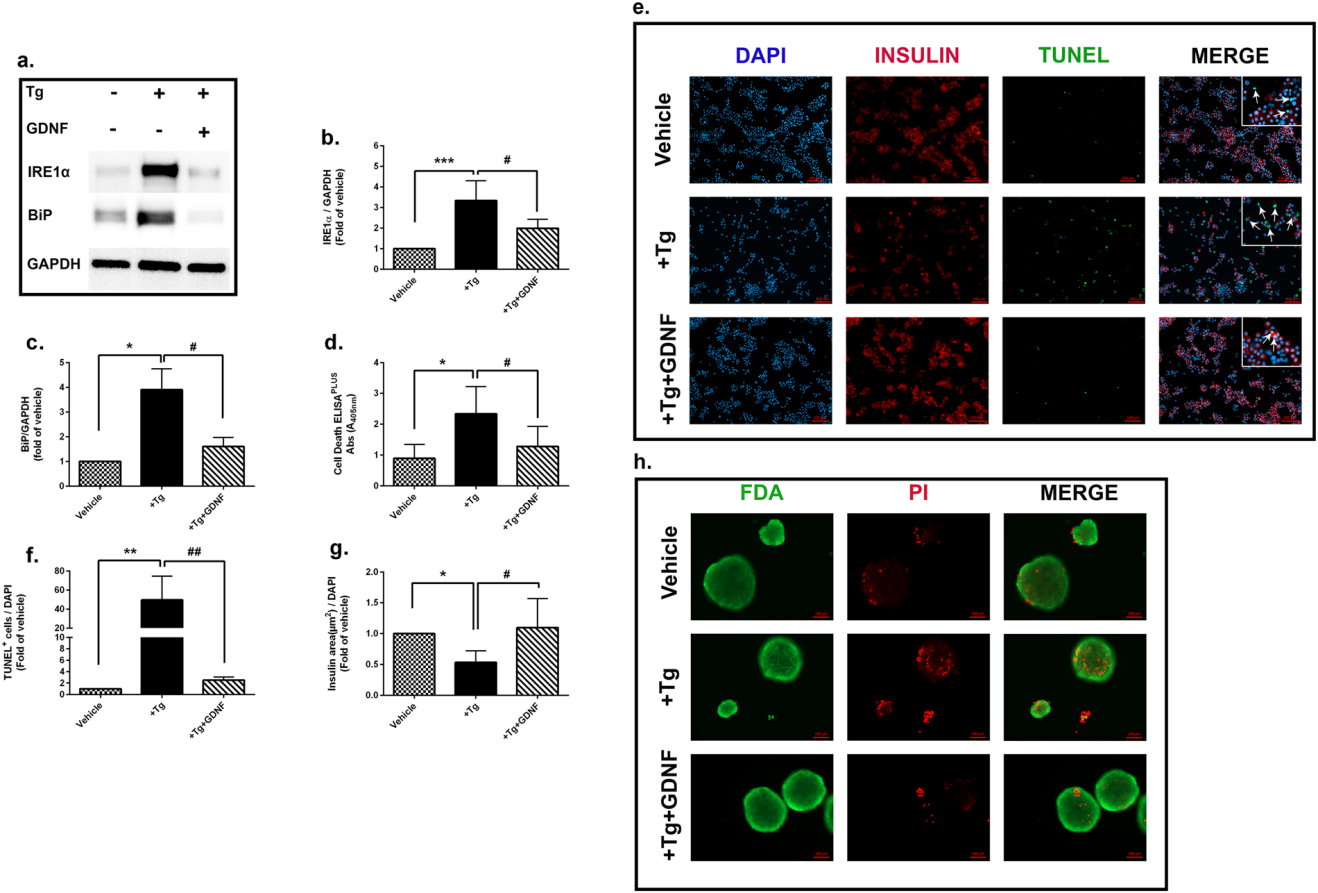
GDNF reduces ER stress and consequently ER stress induced apoptosis in human islets. (**a**) Representative western blot of IRE1α and BiP in human islets treated with Tg (1 µm) with or without GDNF (200 ng/ml) for 48 hrs. (**b**,**c**) Quantification of IRE1α and BiP band densities normalized to housekeeping gene GAPDH. Data is presented as a fold of vehicle islets, n = 6. (**d**) Cell death analysis by cell death ELISA^PLUS^ of human islets treated with Tg with or without GDNF for 48 hrs, n = 9. (**e**) Representative images showing insulin (red), TUNEL (green) and DAPI (blue) nuclear staining of dispersed human islets treated with Tg with or without GDNF for 48 hrs. (**f**) Score of TUNEL^+^ cells (**g**) and measurement of insulin area to DAPI nuclear staining in dispersed human islets. Data is presented as a fold of vehicle islets. n = 3, five images were taken from each slide and minimum of 2000 cells were scored. (**h**) Representative images showing intact islets stained for PI (red) and FDA (green). For all analysis, data is presented as mean ± SD and p-values were analyzed by nonparametric ANOVA with Dunn’s corrections.*p < 0.05, **p < 0.01, ***p < 0.001 vs. vehicle islets, ^#^p < 0.05, ^##^p < 0.01 vs. Tg-treated islets. Tg: Thapsigargin. The n refers to number of independent donors used for each experiment.

It is known that ER stress induces activation of UPR signaling pathways. Prolonged and unresolved activation of UPR leads to apoptosis and loss of beta cells function^5, 29^. Protein folding is highly Ca^2+^-dependent process and depleting the ER-Ca^2+^ stores by blocking sarco/endoplasmic Ca^2+^ -ATPase (SERCA) will thus cause unfolded proteins to accumulate in the ER and subsequently induces ER stress^30, 31^. In order to investigate the effect of GDNF on human islets under ER stress, we cultured nutrient deprived islets with or without GDNF with the SERCA channel blocker, thapsigargin (Tg) for 48 hrs. Tg significantly increased the expressions of the UPR-mediators, IRE1α (3.0 fold of vehicle) and Binding immunoglobulin Protein (BiP) (4.0 fold of vehicle) as determined by western blotting. Importantly, treatment of Tg+GDNF almost completely blunted the upregulation of IRE1α (2.0 fold reduction) and BiP (2.5 fold reduction) compared to the islets treated with Tg alone (Fig. 2a–c). Furthermore, apoptosis measured by DNA fragmentation using Cell Death ELISA^PLUS^ was significantly reduced in Tg+GDNF compared to Tg alone (1.28 ± 0.21vs. 2.3 ± 0.29, p < 0.05) (Fig. 2d). Similarly, immunofluorescent double-staining of dispersed islet cells by TUNEL and insulin (Fig. 2e) showed not only less TUNEL positive cells (Fig. 2e,f), but GDNF also reversed the adverse effect of Tg on insulin staining in human islets (Fig. 2e,g). Lastly, we performed viability staining (FDA/PI) of intact islets and showed enhanced PI staining in the Tg treated islets compared to Tg+GDNF (Fig. 2h). Taken together, these results suggest that GDNF protects human islets from ER stress and consequently ER stress induced apoptosis.

### GDNF reduces ER stress in human islets via PI3K/AKT signaling pathway

**Figure 3:**
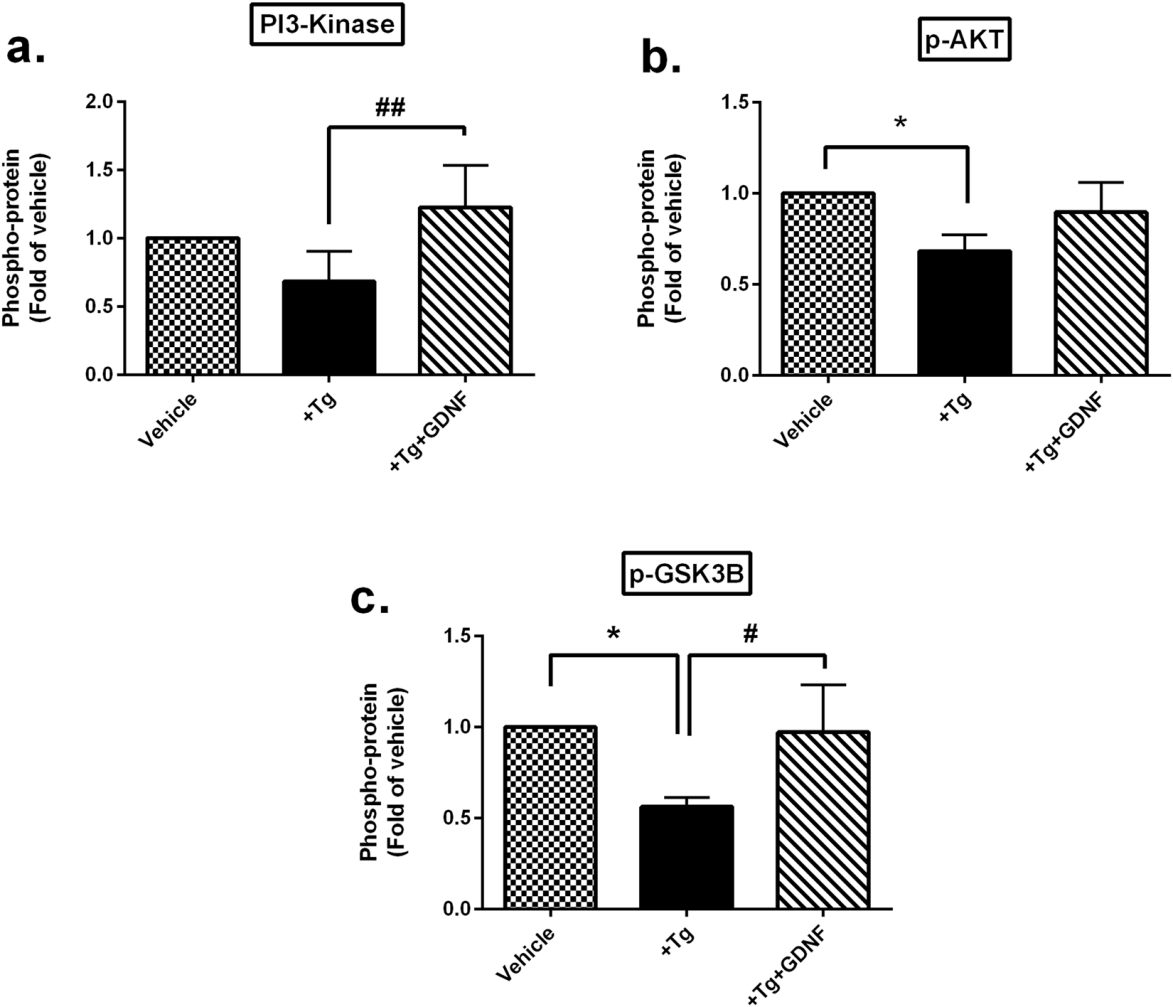
GDNF protective effect on ER stress induced human islets is through activation of PI3K/AKT signaling pathway. (**a–c**) Assessment of phosphoproteins in the PI3K/AKT/GSK3B signaling pathway by multiplex assay in human islets treated with Tg (1 µm) with or without GDNF (200 ng/ml) for 48 hrs. Data is presented as a fold of vehicle islets. n = 6. For all analysis, data is presented as mean ± SD and p-values were analyzed by nonparametric ANOVA with Dunn’s corrections. *p < 0.05vs. vehicle islets, ^#^p < 0.05, ^##^p < 0.01 vs. Tg-treated islets. Tg:Thapsigargin. The n refers to number of donors used for each experiment.

Since ER stress induced apoptosis is partly triggered by reduction of the PI3K/AKT/GSK3B signaling pathway^18^, we investigated the effect of GDNF on phosphorylation of these phosphoproteins in ER stress induced human islets using an intracellular phosphoproteins signaling multiplex assay as described in the methods section. First, Tg treated islets reduced phosphorylation (p-) of PI3K (Fig. 3a), AKT (Fig. 3b) and GSK3B (Fig. 3c) compared to vehicle. Importantly, co-treatment of human islets with Tg+GDNF significantly recovered the levels of p-PI3K, p-AKT, p-GSK3B compared to Tg alone (Fig. 3), suggesting that GDNF protects human islets from ER-stress via activation of the PI3K/AKT signaling pathways.

### Pre-treatment of ER stress induced human islets with GDNF not only improved islets graft function but also alleviated ER stress post transplantation

**Figure 4:**
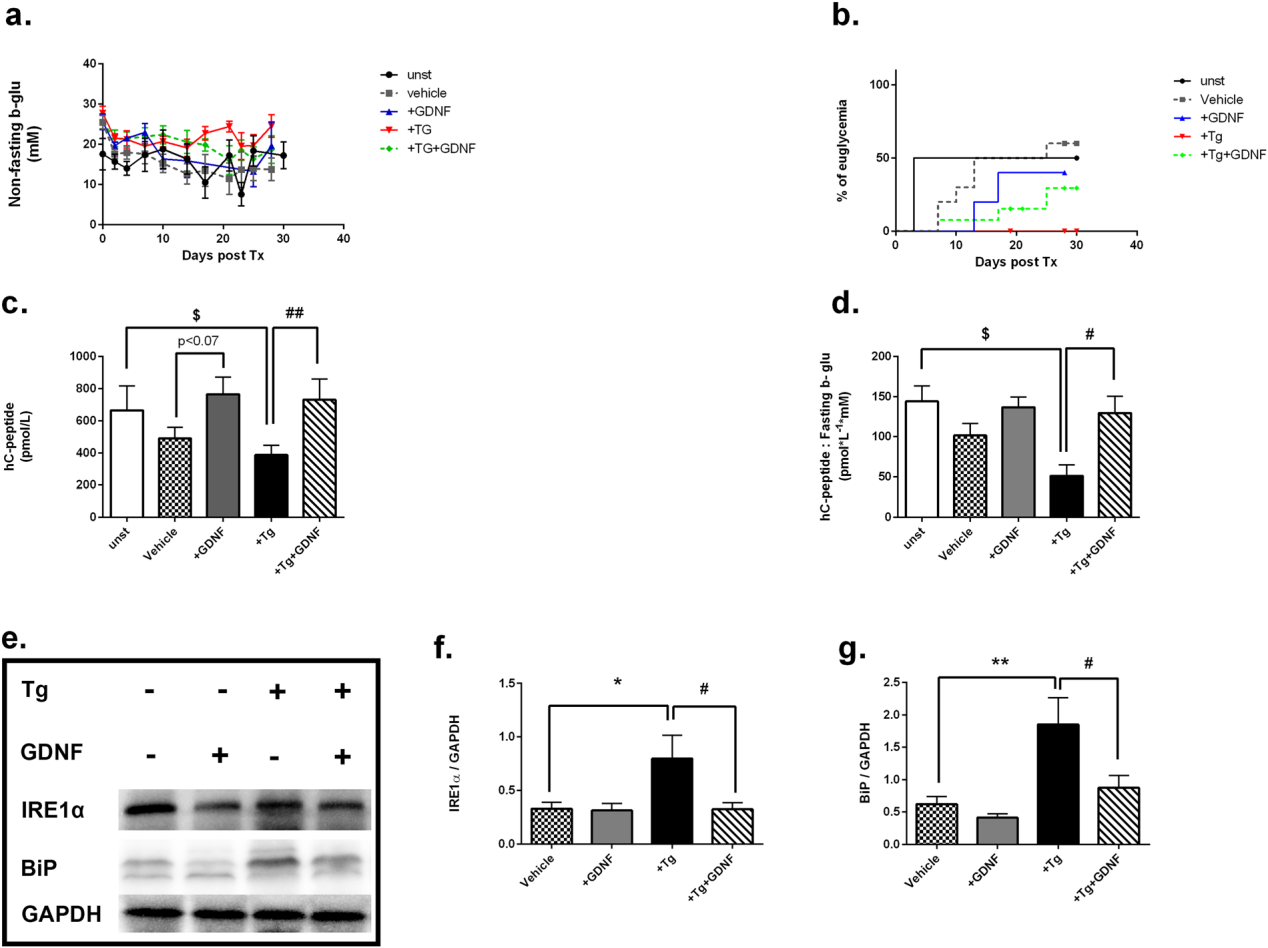
GDNF improves islet grafts function and reduces ER stress post transplantation. (**a**) Random blood glucose levels in diabetic mice transplanted with human islets pre-cultured for 48 hrs in complete medium (unst, n = 6), nutrient deprived medium (vehicle, n = 11) with GDNF (200 ng/ml, n = 6), Tg (1 µM, n = 8), or Tg+GDNF(n = 8). (**b**) Percentage of euglycemic mice post transplantation. (**c**) Circulating levels of hC-peptide measured by EIA in plasma at day 30 post transplantation (**d**) and presented as ratio of hC-peptide over fasting b-glu. (**e**) Representative western blot for protein expression of IRE1α and BiP in human islet grafts harvested on day 30 post transplantation. (**f**,**g**) Quantification of IRE1α and BiP bands densities normalized to housekeeping gene GAPDH. For all analysis, data is presented as mean ± SD and p-values were analyzed by nonparametric ANOVA with Dunn’s corrections or Mann-Whitney U-test. The n refers to number of mice in each experimental group, *P < 0.05, **p < 0.01 vs. vehicle islets, ^#^p < 0.05, ^##^p < 0.01 vs. Tg-treated islets. ^$^p < 0.05 vs unstv group. Log-rank (Mantel-Cox) test was used to analyze the difference in percentage of euglycemic animals post transplantation. Unst: unstarved, Tg: Thapsigargin, B-glu: Blood glucose, hCpeptide: human C-peptide.

Finally, we investigated the possible protective effect of GDNF on nutrient deprived and ER stress induced human islets *in vivo*. Alloxan-induced diabetic male Rag1^−/−^ mice were transplanted under kidney capsule with a minimal dose of human islets (800 islets) pre-cultured for 48 hrs under nutrient deprived culture condition with or without GDNF as well as Tg or Tg+GDNF and followed for 30 days post transplantation. Unstarved islets pre-cultured in complete medium supplemented with 10% human serum prior to transplantation was also included as control. Random blood glucose profile did not shown statistically significant differences among groups (Fig. 4a). Of the animals transplanted with unstarved islets or vehicle, 45–50% became euglycemic on day 3 and between days 7–13 respectively post transplantation. In contrast, none of the diabetic recipient mice transplanted with Tg pre-treated islets reached euglycemia in the follow-up period (Fig. 4b). However, Tg+GDNF recipients showed approximately 25% euglycemia achievement between day 7–17 post transplantation (Fig. 4b). As such, the levels of plasma C-peptide (Tg+GDNF: 814.06 ± 112.04 vs. Tg: 386.07 ± 60.86 pmol/L, p < 0.01) (Fig. 4c), as well as the ratio of human C-peptide to fasting blood glucose (Tg+GDNF: 129.06 ± 20.91 vs. Tg: 51.33 ± 13.70 p < 0.05) (Fig. 4d) were increased at day 30 post transplantation. In addition, GDNF recipients showed a tendency to increased plasma human C-peptide (GDNF: 765.05 ± 107.01 vs. vehicle: 490.06 ± 69.82 pmol/L, p < 0.07) (Fig. 4c) and human c-peptide to fasting blood glucose (136.08 ± 12.56 vs. 101.6 ± 15.09) (Fig. 4d) compared to the vehicle group. We further investigated the influence of GDNF on ER stress in transplanted islet grafts at day 30 post transplantation by immunoblot analysis of ER stress response proteins (Fig. 4e). We found a significantly decrease in protein expression of IRE1α (Fig. 4f) and BiP (Fig. 4g) in grafts containing islets pre-treated with Tg+GDNF compared to Tg alone (2.0 and 3.0 fold reduction, respectively). Taken together, these results suggest that GDNF protects human islets from ER stress and further contributes to improve islet grafts function post transplantation.

## Discussion

In the current study, we have shown that GDNF could reverse the adverse effect of nutrient deprivation and SERCA channel blocker, Tg by improving human islets function and viability through reduction in ER stress induced apoptosis and activation of PI3K/p-AKT/p-GSK3B survival pathway. We have also shown that pre-treatment of nutrient deprived and ER stress induced human islets with GDNF prior to transplantation protected grafts function and mass through a reduction in expression of ER stress mediators.

Strategies to avoid massive beta-cells loss due to islets exposure to hypoxia, nutrient deprivation and activated ER stress during isolation as well as pre- and post- islets transplantation^10, 32–35^ have been of a great interest. In the present study, we mimicked nutrient deprivation and ER stress induced apoptosis by culturing islets under low serum condition and supplementing the ER stress inducer compound, Tg which has been shown previously to increase apoptosis, necrosis and autophagy in both mice and human islets and decreases beta-cell mass^36–38^. Although exposure to the low level of Tg mimics mild ER stress and consequently only disturbs ER Ca^2+^ filing but not islets function and insulin secretion^39^, chronic exposure to elevated level of Tg (1 μM) inhibits insulin secretion as much as 90% and therefore, alleviates islets functionality^40^.

Reducing activated ER stress response has been investigated by supplementing growth factors such as IGF, NGF to islets culture^19, 41, 42^ or through viral delivery of HGF, VEGF and GDNF to islets^20, 43–47^. Co-culturing islets with mesenchymal stem cells which are known as a source of growth factors and cyto-protective elements^48, 49^ have also been studies as a strategy for reducing islets loss. Although elevated islets function and viability as well as improved islets graft revascularization have been shown, most studies are restricted to rodent or non-human primate islets. Recently, a combination of GDNF and IGF have been reported as a beneficial supplement to human islets transplantation medium for improving islets function and viability in culture as well as post transplantation^28^. However, human islets in that study were not exposed to stress responses that normally islets experience in the pre and post transplantation phase. In our study, we show that short-time exposure of human islets to GDNF in culture does not negatively impact human islets viability or functional potency. Importantly, we showed that GDNF recovered insulin secretion in response to stimulated level of glucose and increased total insulin content in nutrient deprived human islets. This was accompanied with a reduction in the ratio of proinsulin to insulin in GDNF-treated human islets. Elevation of proinsulin to insulin ratio reflects failed ER activity in folding newly synthetized proinsulin and processing to insulin^50, 51^. Therefore, reduction in this ratio suggests an overall improvement in insulin process and secretion by GDNF in nutrient deprived islets.

There is a great interest in contribution of ER stress and nutrient deprivation to the failure of functional transplanted islets graft on early phase post transplantation as elevation of ER stress mediators have been reported in isolated and transplanted islets^10, 15, 52^. Herein, we have reported that pre-culturing ER stress-induced human islets with GDNF increased the percentage of euglycemia achievement in diabetic recipients, which was accompanied with significant improvement in human islets function and mass 30 days post transplantation evidenced by increased human C-peptide secretion and human C-peptide to fasting blood glucose ratio, respectively. However, we observed minor protective effect of GDNF compare to vehicle *in vivo*, which could be due to both milder stress response induced by nutrient deprivation alone and shorter culture time in starvation condition prior to transplantation. Improved grafts function post transplantation in animals transplanted with Tg+GDNF pre-treated human islets was associated with significant reduction in expression of ER stress response proteins IRE1α and BiP. Elevations of ER stress sensor, IRE1α and molecular chaperone, BiP have been reported upon ER stress and in pancreatic islets of both type1 and type 2 diabetic patients^5, 53, 54^. An increase in expression of IRE1α is correlated with degradation of insulin mRNA^55, 56^. Therefore, observed reduction in protein expressions of BiP and IRE1α associated with recovered islets mass and function suggest a protective role of GDNF through decreasing protein degradation, reducing ER stress induced apoptosis and also improving ER protein synthesis and folding efficiency. Recently, GDNF has been found as an angiogenic factor secreted by adipose-derived stem cells, which works independent of VEGF and mediates endothelial cells formation and angiogenesis^57^. This could also explain the improvement in transplanted graft function 30 days post transplantation found in our study.

The mechanism involves in the effect of GDNF on pancreatic islets under nutrient deprivation and activated ER stress is not fully understood. Nutrient deprivation induces activation of proinflammatory cytokines in islets^58^. Increased inflammation results in SERCA Ca^2+^ channel instability through activation of oxidative stress and NO production^59, 60^. We induced both nutrient deprivation and ER stress by culturing islets in serum reduced culture medium together with SERCA channel blocker, Tg. Although, we have shown that GDNF reversed the adverse effect Tg through reduction in ER stress activity, it is unknown if GDNF could directly interfere with the SERCA channel or ER Ca^2+^ filing and we could not rule this out in the current study. In addition, GDNF has also been reported to induce protective effect on long-term human islets culture, which is a stress condition independent of nutrient deprivation and inhibition of SERCA channel^28^.

Previous investigations on survival effect of neurotrophic factors such as NGF and GDNF on islets as well as enteric neurons, identified activation of survival pathways PI3K/AKT and glycogen synthase kinase-3β (GSK-3β) as possible candidates involved in cyto-protective effect of these neurotrophic factors^20, 26, 61^. Elevated PI3K/AKT correlates with suppression of ASK, its downstream kinase JNK and therefore reduced apoptosis in human islets^62^. We have demonstrated here that supplementing GDNF to ER stress induced human islets recovered phosphorylation and activation of PI3K, AKT and GSK3β. Therefore, PI3K/p-AKT/p-GSK3β is a possible signaling pathway by which GDNF could protect islets from different stress responses.

In conclusion, by mimicking nutrient deprivation and activated ER stress in isolated islets, we have shown that GDNF could recover human islets function and viability and consequently might be a superior mediator to alleviate stress responses within isolated islets.

## Research Design and Methods

### Human islets isolation and culture

Human islets were obtained from the JDRF award 31-2008-416 (ECIT Islet for Basic Research program) and were isolated according to semi-automated purification system^63^ from male/female 10/8 brain-dead donors with mean age 55 years (19–70 years) provided by the islet isolation facility of the Nordic Network, Uppsala, Sweden, or Oslo University Hospital, Oslo, Norway after appropriate informed consent from relatives for multi-organ donation and for use in research. All experiments and methods using human islets were approved by and performed in accordance with the guidelines and regulations made by regional committee for medical and health research ethics central in Norway (2011/782). Islets purity was judged by digital imaging analysis^64^ or dithizone staining and islets with purity between 50–95% was used in this study. Fresh free floating isolated islets were cultured in CMRL 1066 (Corning, Manassas, VA, USA) containing 10% human serum and supplements as previously described^65^. For experiments, human islets were manually picked and cultured in Sterilin petri dishes (Sterilin LtD, New Port, UK) with CMRL 1066 medium supplemented with 0.5% human AB-serum, 1% penicillin/streptomycin, 10 mM HEPES (Life Technologies AS, Oslo, Norway), with or without human recombinant GDNF (200 ng/ml) (a kind gift from Sven O. Göpel, AstraZeneca R&D, Molndal, Sweden) for 72 hrs at 37 °C (5% CO_2_). In parallel experiments, handpicked human islets were cultured in CMRL medium supplemented with 0.5% human serum and treated with Thapsigargin (Tg) (1 μM) (Sigma Aldrich, Oslo, Norway) with or without GDNF for 48 hrs. Cells and supernatant were harvested as indicated and stored at −80 °C until further analysis.

### Glucose stimulation insulin secretion assay

Ten equally-sized islets were handpicked and transferred into transwells plate (Corning, NY, USA) containing krebs-ringer bicarbonate buffer (1x stock buffer, 1 M Cacl_2_, 1 M Mgcl_2_, 1 M HEPES, 200 mg/ml human albumin) supplemented with 1.67 mM glucose and incubated for 45 min at 37 °C. Transwells were switched to krebs-ringer bicarbonate buffer containing 20 mM glucose and incubated for 45 min at 37 °C. Supernatants were harvested for insulin secretion analysis using human insulin ELISA kit (Mercodia AB, Uppsala, Sweden). Stimulation index (SI) was calculated as a ratio of insulin secreted in high concentration of glucose (20 mM) to insulin in low concentration of glucose (1.67 mM).

### Proinsulin and insulin measurement

Levels of proinsulin and insulin were measured in cell-free supernatant using human insulin and proinsulin ELISA kit (Mercodia AB, Uppsala, Sweden).

### Apoptosis assays

Programmed cell death was analyzed by detection of DNA-histone complexes in the cytoplasmic fraction of islets lysates using Cell Death Detection ELISA^PLUS^ kit (Roche Diagnostics, Mannheim, Germany) according to protocol offered and described by manufacturer.

TUNEL staining using DeadEndTMFluorometric TUNEL system (Promega Biotech AB, Stockholm, Sweden) was performed on 60–80 handpicked equally sized islets. Islets were dispersed into single cells using TrypLE Express (Life Technologies AS, Oslo, Norway) and proceed to universal 320 cyto-centrifuges (Hettich lab technology, Tuttlingen, Germany). Cytospin-made slides were fixed and permeabilized by 4% Paraformaldehyde (PFA) and 0.5% Triton-X100 in PBS respectively. Protein Block Serum Free (DAKO, Oslo, Norway) was used to block non-specific staining. Slides were then incubated overnight at 4 °C with polyclonal Guinea Pig Anti-insulin 1:500 (DAKO, Oslo,Norway). After washing with 1x tris buffered saline plus Tween 20 (TBST), slides were incubated with goat-anti-guinea pig Alexafluor 594 1:300 (Life Technologies AS, Oslo, Norway) for 1 hr at room temperature followed by TUNEL staining according to protocol described by manufacturer. Nuclear staining was performed using SlowFade Gold antifade reagent with DAPI (Life Technologies AS, Oslo, Norway). Images were taken by Axio Observer Inverted Microscope (Carl Ziess AS, Germany) operates by ZEN lite software. Area of insulin positive cells and total number of TUNEL positive cells and nuclei per each image were measured and analyzed using Image J software (National Institute for Health, USA). Five images were taken from each slide and minimum of 2000 cells per slide were scored.

Viability assessment was performed on hand-picked islets using fluorescein diacetate (FDA) 20 µg/ml (Sigma-Aldrich Norway AS, Oslo, Norway) for detection of live cells and propidium iodide (PI) 100 µg/ml (Thremo Fisher Scientific, Oslo, Norway) for evaluating the degree of dead cells. Images were taken by Axio Observer Inverted Microscope (Carl Ziess AS, Germany) operates by ZEN lite software.

### Phosphoproteins analysis of PI3K/AKT signaling pathway

200 handpicked equally sized islets were collected and lysed using cell lysis buffer (BioRad, CA, USA) supplemented with 2 mM PMSF (Sigma Aldrich, Oslo, Norway). Protein lysate concentration was measured by Pierce BCA protein assay (Life Technologies AS, Oslo, Norway) and equal amount of protein lysate was added to each well of phosphoproteins Bio-Plex assay (171V50002M, 171V50007M, 171V500036M). Cell signaling assay was performed according to manufacturer protocol and analyzed using Bioplex 200 system (BioRad, CA, USA).

### Western blot analysis

Cell lysis buffer (RIPA buffer supplemented with Halt protease inhibitor (Thermo scientific, Oslo, Norway) or tissue lysis buffer (RIPA buffer containing halt protease-phosphatase inhibitors and 1% sodium dodecyl sulfate) was added to human islets pellet (100 islets) or frozen graft-bearing kidney samples before proceeding to mechanical disruption using sonication. Samples were centrifuged and purified using QIAshredder purification column (QIAGEN, Hilden, Germany). Total protein concentrations were determined using Pierce BCA protein assay (Life Technologies AS, Oslo, Norway). Equal amounts of total proteins (20 μg) were separated on mini-PROTEIN GTX precast gels followed by proteins bands transfer to PVDF membrane (Bio-Rad, CA, USA). According to antibodies datasheet provided by manufacturer, membranes were blocked with 5% skim milk or 5% BSA in 1xTBST and incubated overnight at 4 °C with primary antibodies, IRE1α rabbit monoclonal antibody 1:1000, BiP rabbit monoclonal antibody 1:1000 (Cell Signaling, MA, USA), GAPDH goat polyclonal antibody 1:1000 (Santa Cruz Biotechnology, TX, USA). Bound antibodies were labeled with goat anti-rabbit IgG-HPR 1:10000 and donkey anti-goat IgG-HPR 1:10000 (Santa Cruz Biotechnology, TX, USA). Protein bands were visualized using clarity western ECL chemiluminescence substrate kit (Biorad, CA, USA) or super signal west femto (Thermo scientific, Oslo, Norway) followed by semi-quantitative measurement of band density using chemiDGC touch imaging system, (BioRad, CA, USA).

### *In vivo* experimental model

The experimental protocol was approved by the Norwegian National Animal Research Authority project license no FOTS id 8588. The animal experiments were performed in accordance with the European Directive 2010/63/EU and The Guide for the Care and Use of Laboratory Animals, 8th edition (NRC 2011, National Academic Press). Animals were housed under standard condition in an approved facility with free access to food and water except fasting time. Male Balb/c Rag 1^−/−^ immunodeficient mice (C.129S7(B6)-Rag1^tm1Mom^/J, stock 003145, The Jackson Laboratory, Sacramento, California, USA) 8–10 weeks old mice were used as recipients. Diabetes was introduced by one intravenously injection of alloxan (Sigma Aldrich, St. Louis, Missouri, USA) (75 mg/kg body weight). Mice constantly showing non-fasting blood glucose above 20 mM for two consecutive days measured by glucometer (Accu-Chek Avia Nano, Rouche Diagnostics, Indiana, USA) were considered diabetic. Mice were divided into five groups and transplanted under kidney capsule with 800 human islets pre-cultured for 48 hrs either in culture medium containing 10% human serum as control (unst) (n = 6), or under nutrient starvation (0.5% human serum)(vehicle)(n = 11) with GDNF (200 ng/ml) (n = 6), Tg (1 µM) (n = 8), or Tg+GDNF (200 ng/ml) (n = 8) as previously described^66^. Random non-fasting blood glucose and weight were monitored every third day at 9 am until endpoint. At day 30 post transplantation, mice under anesthesia were sacrificed by heart puncture for blood samples and the graft-bearing kidney were harvest by snap frozen in liquid nitrogen. Plasma samples for analysis of human C-peptide (Mercodia, Uppsala, Sweden) together with harvested graft-bearing kidneys for analysis of ER stress mediators were stored at −80 C until use.

### Statistical analysis

Data are presented as means ± SD and GraphPad Prism version 6.0. (La Jolla, CA, USA) was used for data analysis. Differences among three groups were evaluated by non-parametric ANOVA with Dunn’s corrections. Mann-Whitney U-test and Wilcoxon matched-pairs test were performed based on experimental design (paired vs. unpaired) for difference analysis between two groups. Significance was set at p < 0.05.
